# The Lc3-synthase gene *B3gnt5 *is essential to pre-implantation development of the murine embryo

**DOI:** 10.1186/1471-213X-8-109

**Published:** 2008-11-12

**Authors:** Franziska Biellmann, Andreas J Hülsmeier, Dapeng Zhou, Paolo Cinelli, Thierry Hennet

**Affiliations:** 1 Institute of Physiology and Zürich Center for Integrative Human Physiology, University of Zürich, Switzerland; 2 Institute of Laboratory Animal Science, University of Zürich, Switzerland; 3 MD Anderson Cancer Center, University of Texas, 1515 Holcombe Blvd, Houston, TX 77030, USA

## Abstract

**Background:**

Glycosphingolipids (GSL) are integral components of mammalian cell membranes that are involved in cell adhesion and cell signaling processes. GSL are subdivided into structural series, like ganglio-, lacto/neolacto-, globo- and isoglo-series, which are defined by distinct trisaccharide cores. The β1,3 N-acetylglucosaminyltransferase-V (B3gnt5) enzyme catalyzes the formation of the Lc3 structure, which is the core of lactoseries derived GSL.

**Results:**

The biological significance of the glycoconjugates produced by the B3gnt5 enzyme was investigated by inactivating the *B3gnt5 *gene in the mouse germline. The disruption of the *B3gnt5 *protein-coding region in mouse embryonic stem cells resulted in reduced Lc3-synthase activity, supporting its specific contribution to lactoseries derived GSL synthesis. Breeding of heterozygous mutant mice failed to produce any viable progeny homozygous for the *B3gnt5*-null allele. The genotypic examination of embryos from heterozygous crosses showed that the disruption of the *B3gnt5 *gene leads to pre-implantation lethality. This finding was compatible with the expression pattern of the *B3gnt5 *gene in the pre-implantation embryo as shown by *in situ *hybridization. The analysis of GSL profiles in embryonic stem cells heterozygous for the *B3gnt5*-null allele confirmed the reduced levels of lactoseries derived GSL levels and of other GSL species.

**Conclusion:**

The disruption of the *B3gnt5 *gene in mice affected the expression of lactoseries derived GLS and possibly of protein-bound β3GlcNAc-linked glycans, thereby demonstrating an essential contribution of these glycoconjugates in early embryonic development, and supporting the importance of these glycoconjugates in cell differentiation and adhesion processes.

## Background

Glycosphingolipids (GSL) represent a large family of glycoconjugates, which are found abundantly on cellular membranes. GSL are classified into different series defined by their respective core structures. In vertebrates, the main GSL series are called ganglio-, lacto-, globo-, isoglobo-, and muco-series [1]. The functional significance of GSL is diverse since these glycoconjugates have been implicated in processes such as cell adhesion, cell migration, regulation of signaling proteins and binding of pathogens and toxins [2,3]. The repertoire of GSL expressed by an organism is subject to changes according to cell type and developmental stage. Consequently, several stem cell and differentiation markers of early embryonic development, such as the stage-specific embryonic antigens SSEA-1, -3 and -4, represent carbohydrate epitopes carried by GSL [4-6].

**Figure 1:**
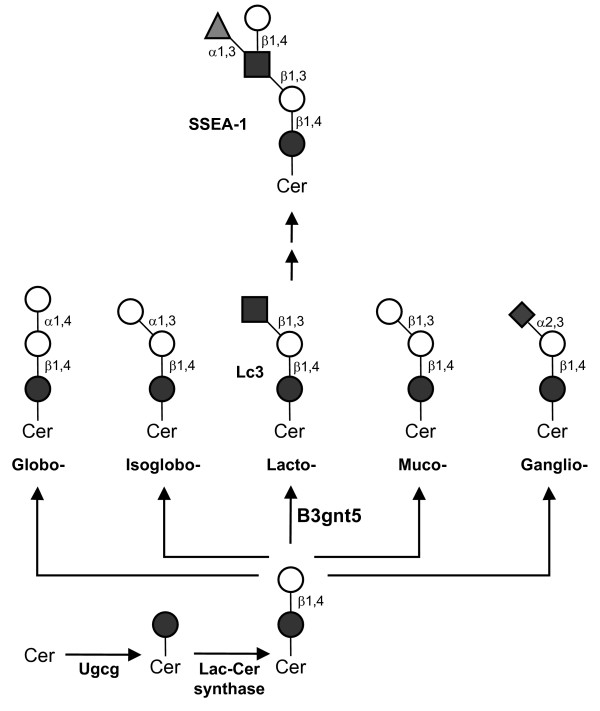
**Biosynthesis of GSL core structures**. GSL biosynthesis is initiated by the transfer of Glc to ceramide (Cer) catalyzed by the Ugcg enzyme. After addition of β1,4-linked Gal by Lac-Cer synthase, distinct core structures are defined by the action of different glycosyltransferases. The B3gnt5 enzyme catalyzes the Lc3 structure, which is the core of lactoseries GSL. The elongation of Lc3 by a α1,3-fucosyltransferase and a β1,4-galactosyltransferase yields the SSEA-1 antigen. ●, Glc; ○, Gal; ■, GlcNAc; ◆ Sia; ▲, Fuc.

The diversity of GSL is shaped by the action of multiple glycosyltransferase enzymes localized in the Golgi apparatus. The inactivation of key glycosyltransferase genes allows investigation of the functional specificity of individual GSL structures or of a whole GSL series. The β1,3 *N*-acetylglucosaminyltransferase-V (B3gnt5) enzyme initiates the formation of the lactoseries GSL by transferring GlcNAc in a β1,3-linkage to lactosylceramide. This transfer event leads to the synthesis of the Lc3 structure (Fig. 1) [7,8]. The *B3gnt5 *gene is expressed during mouse development and then again later mainly in the spleen and placenta in adult mice. Additionally *B3gnt5 *transcripts are found in the adult mouse brain where *B3gnt5 *expression is limited to cerebellar Purkinje cells [7].

The B3gnt5 enzyme has been shown to be key in the expression of sulfoglucuronylglycolipids (SGGL) in the developing nervous system [9]. SGGL are expressed in a regulated manner during embryonic brain development and again postnatally [10-13]. For example, SGGL carry the HNK-1 epitope, which has been implicated in the regulation of synaptic plasticity [14,15]. In addition, the developmentally regulated expression of SGGL coincides with certain cell migration and differentiation phases [16].

The B3gnt5 enzyme also initiates the formation of the SSEA-1 epitope, which is identical to the Lewis X antigen. SSEA-1 corresponds to the trisaccharide Galβ1,4(Fucα1,3)GlcNAc which is first found on 8-cell stage embryos and in mouse embryonic stem (ES) cells [17]. The SSEA-1 epitope, which is mainly found on lactoseries derived GSL, is believed to participate in the regulation of cell adhesion during embryogenesis, cell differentiation, and development of the neuronal system [4,18]. In order to study the developmental and physiological processes mediated by lactoseries derived GSL, we have inactivated the *B3gnt5 *gene in mice by homologous recombination in ES cells. This mouse model suggests an essential contribution of the lactoseries derived GSL series in the very early stages of mouse development.

## Results

**Figure 2:**
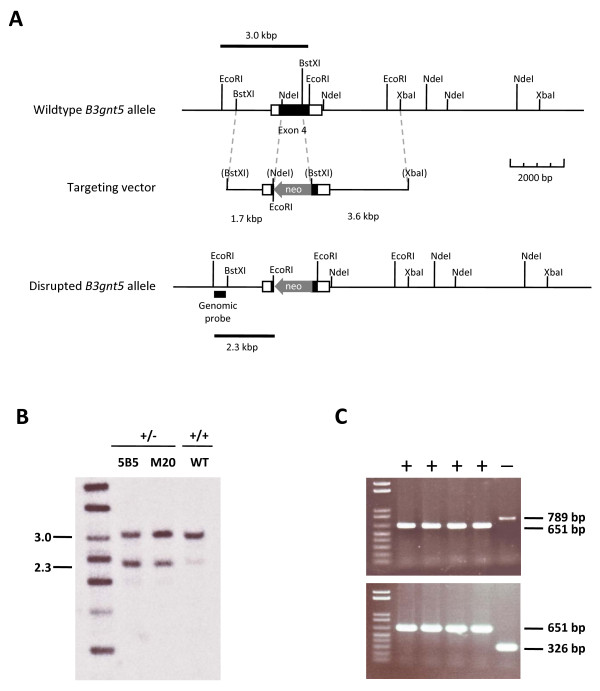
***B3gnt5 *gene targeting in ES cells**. A. The targeting vector was produced by cloning a *neo *resistance cassette into exon 4 of the mouse *B3gnt5 *gene. The construct was flanked by two genomic arms of 1.7 and 3.6 kbp. A genomic probe located just outside of the targeted region was used to detect homologous recombination. B. Genomic Southern blotting of ES cell clones. Genomic DNA of wildtype (WT) ES cells and of two ES cell clones bearing a homologously recombined *B3gnt5 *allele (5B5 and M20) were digested with EcoRI and hybridized to the genomic probe. C. Genotyping of wildtype (+) and targeted (-) *B3gnt5 *alleles by PCR as described in the *Materials and Methods *section.

To address the functional role of the *B3gnt5 *gene in mouse development and physiology, we inactivated this gene by homologous recombination in mouse ES cells. The *B3gnt5 *targeting vector (Fig. 2A) was electroporated into R1 and TC1 ES cells. After selection with G418 and screening of 300 cell clones, we identified four ES cell clones bearing the homologous recombined allele. The homologous recombination at the *B3gnt5 *locus was confirmed using both PCR and genomic Southern blot analysis. The genomic Southern probe distinguished between the wildtype and the null allele by producing diagnostic EcoRI fragments of 3 kbp and 2.3 kbp, respectively (Fig. 2B). The absence of chromosomal aberrations was verified by karyotyping the four homologously recombined ES cells (data not shown).

**Figure 3:**
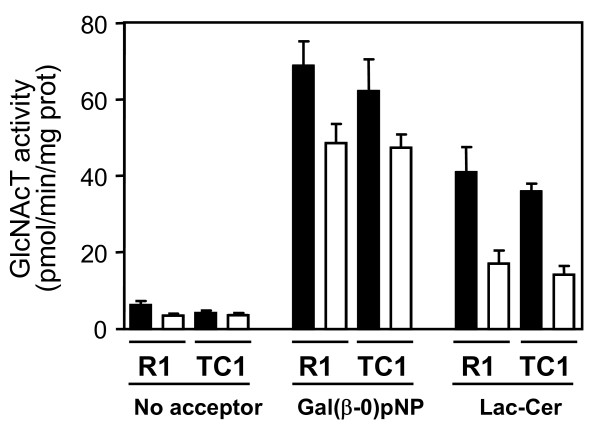
**N-acetylglucosaminyltransferase activity in ES cells**. ES cells of the R1 and TC1 lines were lysed and N-acetylglucosaminyltransferase (GlcNAcT) activity was measured towards the acceptor substrates Gal(β-0)p-nitrophenol (pNP) and lactosylceramide (Lac-Cer). Black bars show GlcNAcT activity of wildtype ES cells and open bars show GlcNAcT activity of *B3gnt5*-targeted ES cells. Each value represents the average + S.D. of three independent measurements.

The inactivation of the *B3gnt5 *gene was confirmed by measuring the level of N-acetylglucosaminyltransferase activity in targeted ES cells. The enzymatic activity towards the artificial acceptor Gal(β1-0)p-nitrophenol and towards lactosylceramide was assayed in wildtype and in *B3gnt5*-targeted ES cells. The transfer of GlcNAc to Gal(β1-0)p-nitrophenol was reduced in R1 and in TC1 *B3gnt5*-targeted ES cells, but did not reached 50% of the activity measured in wildtype cells (Fig. 3). When using lactosylceramide, the physiological acceptor of the B3gnt5 enzyme, the N-acetylglucosaminyltransferase activity detected in the targeted ES cells was indeed reduced to 50% of the normal levels this demonstrating the loss of a functional *B3gnt5 *allele.

The four ES cell clones bearing the *B3gnt5*-null allele were injected into C57BL/6 blastocysts to produce chimeric offspring. Two male chimeric founders were obtained from the blastocysts microinjected with the two R1 ES cell clones, whereas the microinjection of TC1 ES cells did not yield any viable offspring. The chimeric males were mated with wildtype C57BL/6 females and the F1 progeny screened with PCR for germ line transmission of the disrupted allele. Both chimeric males transmitted the null allele to their progeny. Offspring positive for the disrupted gene were backcrossed onto the C57BL/6 background for five generations. All the heterozygous offspring obtained from the chimeric males were grossly normal. Both male and females carriers of the null allele were obtained. Heterozygote mice were then bred to produce homozygous-null offspring. After crossing 17 independent heterozygote breeding pairs, no homozygous-null offspring were detected (Table 1). The ratio of wildtype to heterozygous-null mice was 34% to 66%, thus corresponding to a Mendelian allele distribution assuming a complete lethality of homozygous-null offspring. Since the expression of the *B3gnt5 *gene was shown to be elevated in embryos by mid-gestation [7], we genotyped 69 embryos isolated at E10 to assess whether homozygous-null embryos could be detected. The size and morphology of the embryos isolated at that stage were normal (data not shown) and the genotypic analysis demonstrated that 29.1% and 70.9% of embryos were wildtype and heterozygous-null, respectively (Table 1). Notably, no sites of resorption were observed in the uteri examined at mid-gestation. To identify whether the lack of embryonic development in *B3gnt5*-null mice was related to an implantation defect, we setup time-matings of heterozygous-null mice and examined the genotype of resulting embryos at the blastocyst stage. Again, no homozygous-null embryos were detected (Table 1), thereby indicating that the *B3gnt5 *gene is required for blastocyst development.

**Table 1:** Genotype analysis of *B3gnt5 *heterozygous crosses at various stages of murine development

***Developmental Stage***	***WT (+/+)***	***Het (+/-)***	***KO (-/-)***	**Total screened**
Blastocyst (E3.5)	20.8%	79.2%	0	48
Embryo (E10)	29.1%	70.9%	0	69
Viable pups	34%	66%	0	205

**Figure 4:**
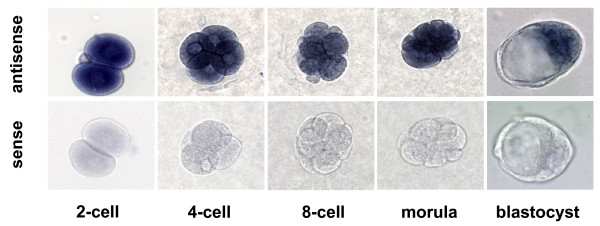
***B3gnt5 *expression in pre-implantation embryos**. All pre-implantation stages investigated show the uniform presence of *B3gnt5 *transcripts up to the morula stage. B3gnt5 transcripts were confined to the inner cell mass at the blastocyst stage. Sense and antisense RNA probes were DIG-labeled as described in the *Materials and Methods *section. The pictures show embryos taken from C57Bl/6 wildtype matings.

Since no homozygous-null embryos could be recovered, we analyzed the expression pattern of *B3gnt5 *in pre-implantation embryos to confirm that the gene was indeed expressed during early embryogenesis. *In situ *hybridization showed the presence of the *B3gnt5 *transcript already at the 2-cell stage. The expression was uniform in all embryonic stages up to the morula stage. In blastocysts, *B3gnt5 *expression was confined to the inner cell mass, as no signal was observed in trophoblast cells (Fig. 4). The expression of the *B3gnt5 *gene throughout early embryogenesis was compatible with the finding of a pre-implantation lethality in *B3gnt5*-null mice.

**Figure 5:**
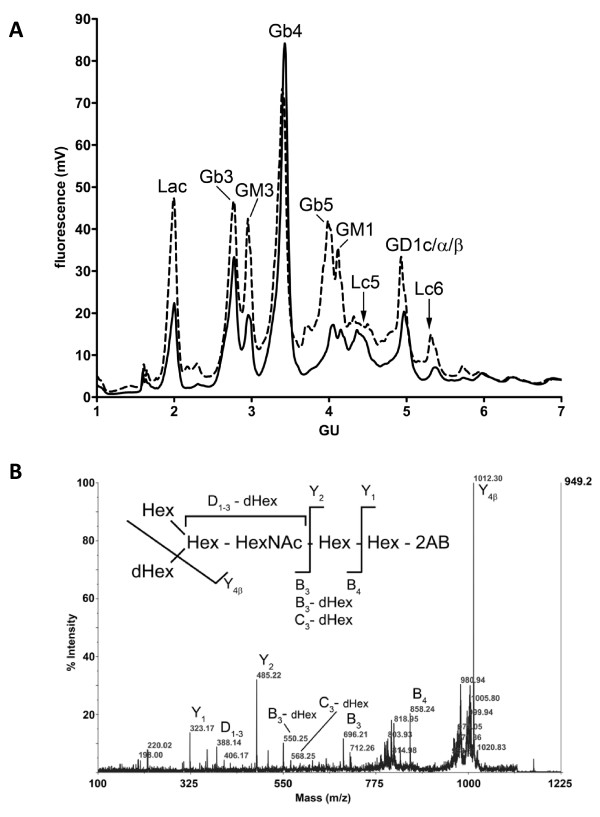
**Glycolipid analysis in ES cells**. A. The oligosaccharide moiety of ES cell GSL was cleaved by ceramide glycanase, labeled with 2-aminobenzamide and separated by normal phase HPLC. The retention times of glucose units (GU) is indicated on the x-axis. The dotted line shows the GSL profile of wildtype R1 ES cells and the plain line shows the GSL profile of the R1 clone 5B5. B. Mass spectrometric fragmentation spectrum of the Lc6 oligosaccharide obtained from the corresponding HPLC fraction. The main fragment ions are labeled and their assignment to the Lc6 structure is depicted.

The inner cell mass of blastocysts is the source of ES cells used for gene targeting. Accordingly, we examined the level of *B3gnt5 *gene expression in the R1 and TC1 ES cells by quantitative PCR. The *B3gnt5 *mRNA levels were identical in the two cell lines and comparable to the expression of the housekeeping *Polr2a *polymerase gene (data not shown). Because the *B3gnt5 *enzyme is involved in the biosynthesis of the lactoseries derived GSL, we also analyzed GSL profiles quantitatively in wildtype and in *B3gnt5*-targeted R1 and TC1 ES cells. GSL were isolated from ES cells by organic solvent extraction and digested by ceramide glycanase to release the carbohydrate moiety. The resulting oligosaccharides were labeled with 2-aminobenzamide at their reducing end and separated by HPLC [19]. The comparison between wildtype and *B3gnt5*-targeted ES cells showed decreased amounts of several GSL structures (Fig. 5A). The lactoseries GSL Lc3, the product of the *B3gnt5 *enzyme, was not detected in ES cells. However, its elongation products, the lactoseries derived Lc5 and Lc6 were identified and found to be present at lower levels in *B3gnt5*-targeted ES cells. The decreased amounts of GSL found in *B3gnt5*-targeted ES cells were not limited to lacto-series GSL, since the gangliosides GM3, GM1, GD1 and the globosides Gb3, Gb5 were also reduced. The identity of the oligosaccharide structures detected by HPLC was confirmed by mass-spectrometry. The oligosaccharide sequences were further examined by fragmentation analysis (Fig. 5B). This analysis of GSL profiles on ES cells demonstrated the contribution of the *B3gnt5 *enzyme to the formation of lactoseries derived GSL.

## Discussion

By disrupting the *B3gnt5 *gene, we aimed to improve current appreciation of the functions of lactoseries derived GSL *in vivo*. Our study suggests an essential role of this GSL series in pre-implantation embryonic development by showing that *B3gnt5*-null blastocysts could not be retrieved from heterozygous matings. The expression of the *B3gnt5 *gene from the two-cell stage up to the blastocyst stage would suggest a role for lactoseries derived GSL in cell-cell adhesion, possibly contributing to embryo compaction and implantation. Similarly, the SSEA-1 antigen has been shown to mediate the tight adhesion of blastomeres, since the addition of multivalent SSEA-1 structures promotes embryo decompaction [20]. The formation of the SSEA-1 antigen requires the action of the α1,3 fucosyltransferase FUT9 [21]. The disruption of the *Fut9 *gene in mice abolished the formation of the SSEA-1 antigen in pre-implantation embryos, but this loss did not affect the compaction and implantation processes [18]. This finding showed that the fucose residue is critical for marking the antigen but that fucose is dispensable for the potential adhesion properties of the lactoseries derived structure.

The early lethality observed in the *B3gnt5 *gene disruption model contrasts with the phenotype described for mice lacking the ceramide glucosyltransferase *Ugcg *gene, which initiates GSL biosynthesis (Fig. 1). *Ugcg *homozygous-null embryos have been reported to die between embryonic days E7.5 and E9.5 [22]. Since Ugcg activity is required for the formation of lactoseries derived GSL, the reverse order would be expected for the onset of lethality in the *Ugcg *and *B3gnt5 *gene disruptions. A similar paradoxical situation was observed in the globoside GSL pathway when comparing the phenotypes resulting from the disruptions of the α1,4 galactosyltransferase Gb3-synthase [23] and β1,3 N-acetylgalactosaminyltransferase Gb4-synthase [24] genes. Mice lacking the Gb3-synthase gene developed normally and did not show any physiological abnormality [25]. By contrast, the disruption of the Gb4-synthase gene arrested development at the blastocyst stage and prevented the implantation of mouse embryos [26]. This apparent discrepancy may have different causes. It is possible that the loss of Ugcg activity is compensated for by other glycosyltransferase activities expressed during pre-implantation stages. However, no glycosyltransferase has been described with such activity yet and no proteins similar to the Ugcg enzyme could be retrieved from the mouse genome so far. Alternatively, it is possible that the expression of the *Ugcg *gene in unfertilized oocytes [27] yields enough of the Ugcg enzyme to sustain the formation of Glc-Cer until gastrulation.

The enzymatic characterization of the B3gnt5 protein has shown a significant N-acetylglucosaminyltransferase activity towards the monosaccharide Gal [7]. Therefore it is conceivable that B3gnt5 may act on Gal-termini found on N- or O-linked glycans. However, it is likely that pre-implantation lethality is in fact mediated by a defect of GSL biosynthesis, since defects in N- or O-linked glycan elongations lead to post-implantation lethal phenotypes at the earliest [28]. Indeed, the inactivation of the B3gnt2 gene, a paralog of B3gnt5 that mediates the formation of poly-N-acetyllactosamine chains on glycoproteins, does not impair embryonic development [29].

Even if the pre-implantation lethality observed in the *B3gnt5*-null embryos is caused by a defect of GSL biosynthesis, the question remains whether the loss of *B3gnt5 *solely affects the lactoseries derived GSL pathway. Our analysis of GSL profiles in ES cells bearing a *B3gnt5*-null allele showed decreased levels of additional GSL structures, such as the gangliosides GM3, GM1, GD1 and the globosides Gb3, Gb5 (Fig. 5). Earlier work has documented the physical interaction of glycosyltransferases involved in GSL biosynthesis [30], suggesting that the loss of a single enzyme might destabilize the localization and thereby the activity of other enzymes.

## Conclusion

The disruption of the *B3gnt5 *gene leads to the earliest lethality reported for a Golgi-localized glycosyltransferase, thereby underlining the essential role of lactoseries derived GSL and possibly of protein-bound β3GlcNAc-linked glycans in pre-implantation embryogenesis. Because of this early lethality, the role of these glycoconjugates in subsequent developmental pathways could not be determined in the present model. The expression pattern of the *B3gnt5 *gene at gastrulation and at later stages would support the involvement of *B3gnt5 *products in processes such as neurogenesis and brain development. The production of a conditional *B3gnt5 *gene targeting model will certainly bring new insights on the role of lactoseries derived GSL in organ development and functions.

## Methods

### *B3gnt5 *gene targeting

The targeting vector was assembled by flanking the PGK-Neo cassette of the pPGK-Neo plasmid [31] with two fragments of 129Sv/J genomic DNA fragments isolated from a λFIX-II bacteriophage library (Stratagene). The left arm, a 1.7-kbp BstXI-NdeI fragment that includes 400 bp of the *B3gnt5 *exon 4, was inserted blunt-end into the NotI site of pPGK-Neo and the right arm of the targeting vector was subcloned blunt-end as a 3.6-kbp BstXI-XbaI fragment into the EcoRV site of pPGK-Neo. Using this construct, a stretch of 745 bp encoding the catalytic domain of *B3gnt5 *was replaced by the PGK-Neo cassette (Fig. 2A). The targeting vector was linearized at the unique Sac II site and 10 μg were electroporated into 5 × 10^6 ^mouse embryonic stem (ES) cells of line R1 [32] and TC1 [33]. Cells were seeded on gelatin-coated Petri dishes and cultured in KO DMEM (Gibco) containing 15% fetal calf serum (Gibco), 1000 U/ml leukemia inhibitory factor (ESGRO, Gibco) and G418 (200 μg/ml). After 5 days of selection, 300 clones were picked and tested for homologous recombination by PCR and genomic Southern blotting.

### Genotyping of ES cell clones

Homologously recombined *B3gnt5 *alleles were identified by PCR amplification of a 1920-bp fragment comprising the boundary of PGK-Neo up to the genomic DNA proximal to the left arm of the targeting vector. The PCR reaction was carried out using 50 ng of genomic DNA with the primers 5'-TACTACCCTGTCTAGGAGCAGTTG-3' and 5'-CATCGCATTGTCTGAGTAGGTGTC-3' for 35 cycles at 94°C for 45 s, 52°C for 1 min, and 72°C for 2 min. Homologous recombination at the *B3gnt5 *locus was confirmed by genomic Southern blotting. Genomic DNA (5 μg) samples were digested with EcoRI transferred to Hybond-N membranes (GE Healthcare) and hybridized to a 530-bp EcoRI-BstXI genomic fragment (Fig. 2A) as a probe. The detection of a 2.3-kbp fragment was indicative of a targeted *B3gnt5 *allele, whereas the wildtype allele was detected as a 3.0-kbp fragment.

### Generation and breeding of *B3gnt5*-targeted mice

The homologously recombined ES cell clones were karyotyped using the standard potassium chloride method [34]. ES cell clones harboring 40 chromosomes were injected into blastocyst-stage embryos. The resulting chimeric males were bred at 8 weeks of age with C57BL/6 females and germ-line transmission was observed with the birth of agouti offspring. Mice harboring the targeted *B3gnt5 *allele were backcrossed for five generations to the C57BL/6 background.

### Genotyping of mouse samples

The *B3gnt5*-wildtype and -null alleles were detected in DNA isolated from tail biopsies, from embryonic-day-10 (E10) whole embryos and from blastocyst-stage embryos. Blastocysts were harvested from time-mated pregnant females by flushing the uterine horns with M2 buffer (Sigma). The tissue samples were digested with a 25 μg/ml proteinase K solution at 56°C for 16 h and the reaction was stopped by incubation at 95°C for 10 min. The wildtype allele was detected by PCR using the primers 5'-GGCTCAAGATGTCCTCCTCTTA-3' and 5'-ACATGGTCCTGTGGCAAGATTC-3' that yielded a 651-bp fragment. The null allele was detected as a 789-bp PCR fragment using the primers 5'-ACTCGTCAAGAAGGCGATAGAA-3' and 5'-CGGCCATTGAACAAGATGGATT-3'. These PCR reactions were both run for 35 cycles at 94°C for 1 min, 60.5°C for 45 s, and 72°C for 1 min. Another PCR protocol was applied to detect the *B3gnt5*-null allele in blastocysts. The primers 5'-CATCAGCCGCTACAGTCAAC-3' and 5'-CATCAGAGCAGCCGATTGTC-3' yielded a 326-bp fragment corresponding to a fragment of the PGK-Neo cassette. The corresponding PCR conditions were 35 cycles at 94°C for 45 s, 63.5°C for 40 sec, and 72°C for 40 s.

### Glycosyltransferase activity assays

N-acetylglucosaminyltransferase activity was assayed in *B3gnt5*-targeted ES cells as described previously [7]. ES cells (1 × 10^7^) were released by trypsin digest, washed in PBS twice and lyzed in 200 μl of 2% Triton X-100 in 50 mM cacodylate buffer, pH 7.0, 20 mM MnCl_2 _for 15 min on ice in presence of a protease-inhibitor cocktail (Complete EDTA-free, Roche). Reactions were run at 37°C for 4 h using 25 μl of post-nuclear supernatant in 50 μl reactions of 50 mM cacodylate buffer, pH 7.0, 20 mM MnCl_2_, 5% Me_2_SO, 0.75 mM ATP, 0.5 mM UDP-GlcNAc including 5 × 10^4 ^cpm of UDP- [^14^C]GlcNAc (GE Healthcare). Reactions were stopped by adding 500 μl ice-cold H_2_O. The reaction products were purified by C18 SepPak cartridges (Waters) and measured by scintillation counting [35].

### Isolation of pre-implantation embryos

Pre-implantation embryos were obtained from superovulated females. Superovulation was carried out by intraperitoneal administration of 50 IU pregnant mare serum gonadotropin (PMSG) (Intervet, Veterinaria AG Zürich, Switzerland) and 25 IU human chorionic gonadotropin (hCG) (Intervet) 48 h later [36]. Embryos were removed at E1.5 (2–4-cell stage) and E3.5 (blastocyst stage). To obtain embryos at the 4-cell up to the morula stage, 2-cell embryos were cultivated in M16 media (Sigma) until the desired stage was reached. E3.5 blastocysts were isolated by flushing of the uterine horn.

### *In Situ *Hybridization

Embryos were fixed for 1 h at room temperature in 4% paraformaldehyde in PBS and washed twice in PBS/0.1% Tween (PBT) and dehydrated once in 25%, 50%, 75% and twice in 100% methanol/PBT. The dehydration was followed by rehydration in the reverse order of the MeOH/PBT series 75%, 50%, 25% for 5 min each. The embryos were permeabilized in RIPA buffer and refixed in 4% PFA/0.2% glutaraldehyde. Embryos were washed in a 1:1 mixture of PBT/hybridization solution (50% formamide, 5× SSC, 0.1% Tween-20, 0.1% SDS, 50 μg/ml E. coli tRNA, 60 mM citric acid) for 10 min and then in hybridization solution. *In situ *hybridization was performed as described previously [37]. As a control for the specificity of the labelings in each hybridization experiment, control embryos were hybridized with an equal concentration of a sense probe transcribed from the same template as the antisense probe. After staining over night at 4°C, embryos were post fixed in 4% paraformaldehyde/0.1% glutaraldehyde in PBT for 1 h and washed twice in PBT, then cleared in glycerol: PBT (1:1) and stored in glycerol: 2 mM EDTA in PBT (4:1). Hybridization results were documented using a Zeiss Axiovert 200 M microscope (Carl Zeiss AG, Feldbach, Switzerland). The *B3gnt5 *908-bp riboprobes were prepared as described previously [7]. T7 and SP6 riboprobes were made using a DIG RNA labeling kit (Roche, Switzerland) and alkaline hydrolyzed to reduce the size of the riboprobes to about 300 bp.

### Glycosphingolipid analysis

GSL were extracted from mouse ES cells three times with chloroform:methanol:water (4:8:3, v:v:v) [38]. The extracts were pooled, dried under N_2 _and re-dissolved in chloroform:methanol:PBS (1:163:160, v:v:v). A 1 ml SepPak tC18 cartridge (Waters) was conditioned with 2 ml methanol and 2 ml water. The dissolved lipid extract, corresponding to 50 mg wet weight of cell pellets were applied to the SepPak tC18 cartridge, followed by washing with 3 ml water. GSL were eluted with 1 ml chloroform:methanol (98:2, v:v), 2 ml chloroform:methanol (1:3, v:v) and 1 ml methanol [39]. Eluates were dried under N_2 _and subjected to ceramide glycanase digestion, 2-aminobenzamide-labelling and NP-HPLC analysis as described earlier [40]. One minute fractions were collected from the NP-HPLC and aliquots were subjected either directly or after desalting with 100 μl custom packed graphite columns (ENVI-Carb, Supelco) to MALDI-MS.

### Mass Spectrometry

The MALDI matrix was prepared by suspending 10 mg DHB in 1 ml of 50% acetonitrile, containing 1 mM NaCl. Sample and matrix were mixed on the MALDI plate at a ratio of 1:1 and allowed to dry at room temperature. The dried spots were re-crystallized by applying < 0.1 μl ethanol. MALDI mass spectra were recorded in positive ion mode, using an Applied Biosystems 4800 Proteomics Analyzer (Applied Biosystems). Averages of 2000 to 5000 laser shots were used to obtain MS/MS spectra. The collision energy was set at 1 kV and the air pressure inside the collision cell was set at 2 × 10^-6 ^Torr.

## Authors' contributions

FB screened ES cells for homologous recombination characterized positive ES cell clones, was responsible for mouse breeding and phenotyping, and participated to manuscript preparation. AJH extracted and analyzed GSL profiles in ES cells and participated to manuscript preparation. DZ constructed the *B3gnt5 *targeting vector, characterized homologous recombinant ES cell clones and participated to manuscript preparation. PC assisted FB and contributed to embryo isolation, fixation and in situ hybridization, and participated to manuscript preparation. TH initiated and directed the study, participated to the genotype analysis of mouse embryos, and wrote most of the manuscript. All authors read and approved the final manuscript.
